# Effects of Plasmin on von Willebrand Factor and Platelets: A Narrative Review

**DOI:** 10.1055/s-0038-1660505

**Published:** 2018-06-07

**Authors:** Lisa N. van der Vorm, Jasper A. Remijn, Bas de Laat, Dana Huskens

**Affiliations:** 1 Synapse Research Institute, Maastricht, The Netherlands; 2 Cardiovascular Research Institute Maastricht, Maastricht University Medical Centre, Maastricht, The Netherlands; 3 Department of Clinical Chemistry and Hematology, Gelre Hospitals, Apeldoorn, The Netherlands

**Keywords:** plasmin, von Willebrand factor, platelets

## Abstract

Plasmin is the major fibrinolytic protease responsible for dissolving thrombi by cleavage of its primary substrate fibrin. In addition, emerging evidence points to other roles of plasmin: (1) as a back-up for ADAMTS13 in proteolysis of ultra-large von Willebrand factor (VWF) multimers and (2) as an activator of platelets. Although the molecular mechanisms of fibrinolysis are well defined, insights on the effects of plasmin on VWF and platelets are relatively scarce and sometimes conflicting. Hence, this review provides an overview of the literature on the effects of plasmin on VWF multimeric structures, on VWF binding to platelets, and on platelet activation. This information is placed in the context of possible applications of thrombolytic therapy for the condition thrombotic thrombocytopenic purpura.

## Introduction

Plasmin is the key protease of the fibrinolytic system. During clot development, fibrin deposition elicits generation of plasmin from plasminogen, resulting in the dissolution of the fibrin clot. 1 Although many studies have focused on mechanisms of plasmin-mediated fibrin cleavage (reviewed by, among others, Cesarman-Maus and Hajjar 2 ), there is a relative gap in knowledge on the effects of plasmin on other components that form the primary blood clot/thrombus. Recently, there has been increasing attention for a potential role of plasmin in the treatment of disorders associated with spontaneous von Willebrand factor (VWF)–platelet aggregation, such as thrombotic thrombocytopenic purpura (TTP). 3 4 This review provides an overview of the literature on the effects of plasmin on VWF and platelets. After a brief introduction into the structure and functions of plasmin, the evidence supporting a role for plasmin in degradation of VWF multimers and VWF–platelet complexes is presented. This is followed by a summary of existing literature on the effect of plasmin on platelet activation through the platelet GP1b, GPIIb/IIIa, and thrombin receptors.

## Plasmin Function and Regulation

**Fig. 1:**
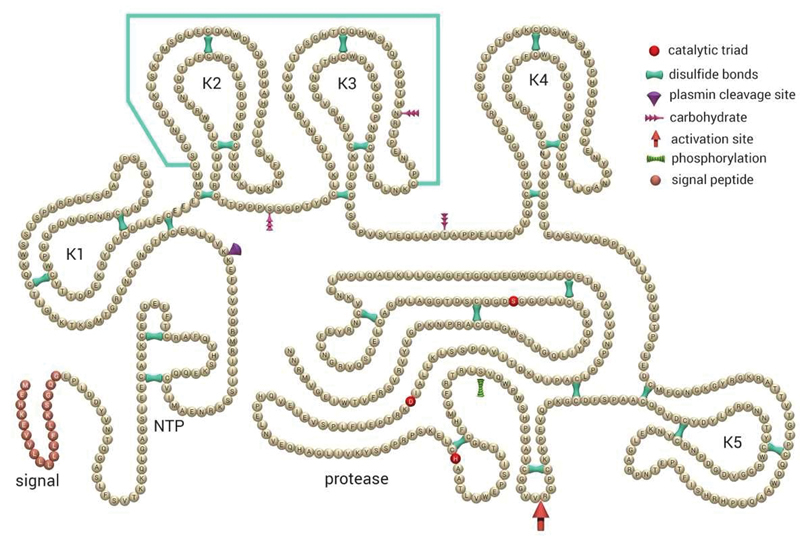
Schematic representation of the primary structure of human plasminogen. The catalytic triad (His603, Asp646, and Ser741) within the protease domain, the activation site (Arg561–Val562), and the 24 disulfide bridges, as well as the signal peptide are indicated. NTP N-terminal peptide; K1–K5 kringles 1–5. (Adapted from Schaller and Gerber. 100 )

Plasmin is a potent serine-protease that is generated from its zymogen, plasminogen, usually at sites of vessel damage. Synthesized primarily in the liver, 5 plasminogen circulates in plasma at a concentration of approximately 1.5 μmol/L, with a half-life of approximately 2 days. 6 Native, circulating Glu-plasminogen (92 kDa) is a single chain glycoprotein with a glutamic acid (Glu) as the N-terminal residue, containing an N-terminal activation peptide (NTP), five homologous triple-loop structures called kringle domains (K1–K5), and the serine protease domain containing the catalytic triad ( Fig. 1 ). 7 Physiological activation of plasminogen is mediated by tissue-type plasminogen activator (tPA) and urokinase-type plasminogen activator (uPA, urokinase). 6 tPA and uPA specifically cleave the single activation bond Arg561–Val562 of plasminogen, resulting in the formation of the two-chain enzyme plasmin composed of an N-terminal heavy chain (12–65 kDa) and a C-terminal light chain (25 kDa). 8 Activation of plasminogen is inhibited by plasminogen activator inhibitors types 1 and 2 (PAI-1 and PAI-2). 9 10

**Fig. 2:**
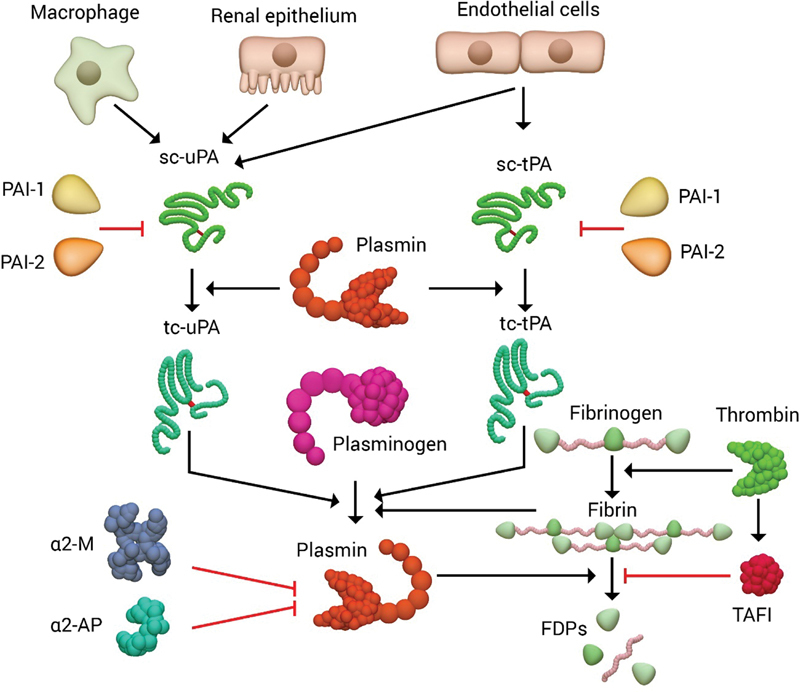
Overview of fibrinolysis. Single-chain (sc) tPA and uPA are secreted from endothelial cells and from renal epithelium, monocytes/macrophages, or endothelial cells, respectively. Both tPA and uPA can be inhibited by plasminogen activator inhibitors (PAI-1 and PAI-2). Once plasmin is generated, it converts single-chain tPA and uPA to two-chain (tc) forms. Plasminogen is converted into the active protease plasmin primarily by tc-tPA (with fibrin as a cofactor) or tc-uPA. Plasmin cleaves fibrin to fibrin degradation products (FDPs), which can be inhibited by TAFI. Plasmin itself is inhibited by α2-antiplasmin (α2-AP) and α2-macroglobulin (α2-M).

The primary substrate of plasmin is fibrin, which regulates its own degradation by binding both plasminogen 11 and tPA 12 on its surface, thereby localizing and enhancing plasmin generation. This binding of plasminogen to fibrin can be blocked by thrombin-activatable fibrinolysis inhibitor (TAFI). 13 Importantly, plasmin can also cleave both tPA and uPA, transforming them from single chain to more active two-chain polypeptides, thereby forming a positive-feedback loop ( Fig. 2 ). 2 Plasmin is rapidly inhibited by its major inhibitor α2-antiplasmin (α2-AP), and to a lesser extent by α2-macroglobulin (α2M), unless it remains bound to fibrin or to its cell surface receptors ( Fig. 2 ). 14

Plasmin does not only cleave fibrin, it also possesses exceptionally broad specificity for target substrates, targeting among others coagulation factors V, 15 VIII, 16 IX, 17 X, 18 TFPI, 19 and fibrinogen; 20 as well as the VWF cleaving protease ADAMTS13 (a disintegrin and metalloproteinase with a thrombospondin type 1 motif, member 13) 21 ( Table 1 ).

**Table 1:** Plasmin substrates and corresponding cleavage sites: amino acid residue preceding cleavage site is given

Substrate	Plasmin cleavage site	Reference
Fibrin(ogen)	Arg104 (α chain) Arg42 (β-chain) Lys133 (β-chain) Lys62 (γ-chain) Lys85 (γ-chain)	Walker and Nesheim 101
Glu-plasminogen	Lys62 Arg68 Lys77	Wiman 102 Wiman and Wallén 103 Violand and Castellino 104
Factor V	Arg348 Lys1656 Arg1765 Lys1827	Omar and Mann 15 Lee and Mann 105 Zeibdawi and Pryzdial 106
(sc-)tPA	Arg275	Pennica et al 107 Johanessen et al 108
(sc-)uPA	Lys158 Lys135	Irigoyen et al 109 Collen and Lijnen 20
TFPI	Lys86 Arg107 Arg199 Lys249 Lys256	Li and Wun 19
Factor X	Lys433	Pryzdial et al 18
Factor IX	Lys43 Arg145 Arg180 Lys316 Arg318	Samis et al 17
TAFI	Arg92 Arg302 Lys327 Arg330	Marx et al 110
Factor VIII	Lys36 Arg336 Arg372 Arg740	Nogami et al 111 Ogiwara et al 16 Nishiya et al 112
ADAMTS13	Unknown	Crawley et al 21
VWF	Lys1491	Brophy et al 3

Abbreviations: ADAMTS13, a disintegrin and metalloproteinase with a thrombospondin type 1 motif, member 13; sc, single-chain; TFPI, tissue factor pathway inhibitor; tPA, tissue plasminogen activator; VWF, von Willebrand factor.

## Effect of Plasmin on von Willebrand Factor

### Structure and Regulation of von Willebrand Factor

VWF is a large plasma glycoprotein with essential functions in hemostasis. VWF circulates in plasma, at a concentration of approximately 10 μg/mL, 22 in the form of multimers that comprise a varying number of VWF monomers (250–270 kDa). 23 Thus, the molecular weight of VWF multimers ranges from 500 to 20,000 kD, depending on the number of subunits. 24 Each VWF monomer possesses several domains, namely, the D'D3, A1, A2, A3, D4, C1 to C6, and CK domains. 25 Under normal blood flow conditions, VWF adopts a globular conformation 26 that can bind coagulation FVIII, prolonging its lifetime in the circulation. 27 28

**Fig. 3:**
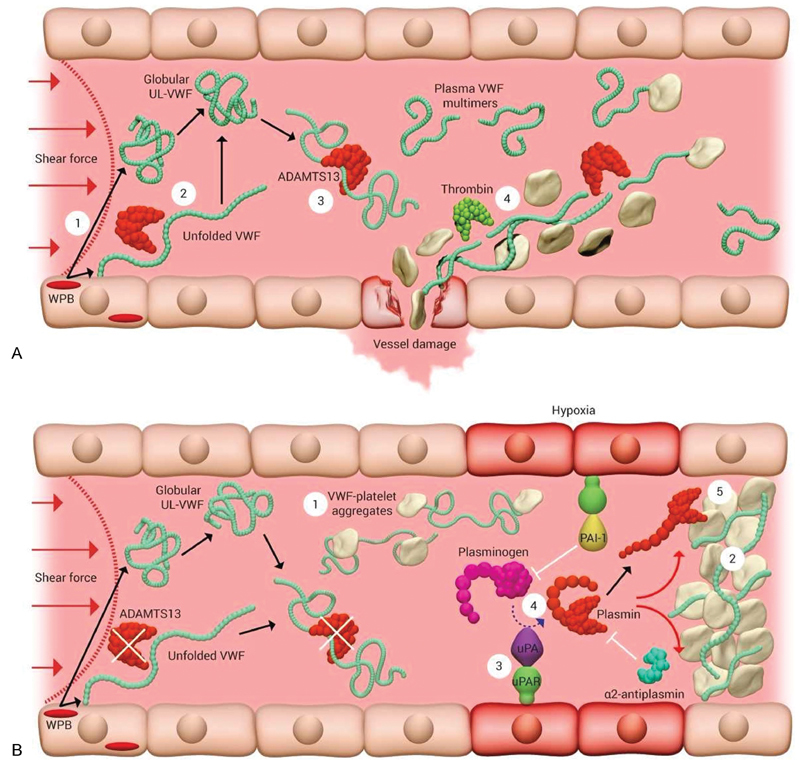
Model of VWF cleavage by ADAMTS13 ( **A** ) and plasmin ( **B** ). ( **A** ) Graphic representation of VWF proteolysis by ADAMTS13. (1) UL-VWF in a globular conformation is synthesized by the endothelium, stored within, and secreted into the circulation by Weibel-Palade bodies (WPB). (2) Alternatively, a proportion of UL-VWF may remain transiently bound to the endothelial surface during exocytosis and unfold to a stretched conformation in response to shear. This conformation exposes the VWF A2 domain, enabling ADAMTS13 to cleave and release VWF into the circulation, where it adopts a globular conformation. (3) During passage through the microvasculature, globular UL-VWF in free circulation may unravel partially, allowing for cleavage of the largest, most thrombogenic multimers to smaller multimers that do not spontaneously interact with platelets. (4) At sites of vessel damage, plasma VWF will bind to exposed subendothelial collagen and subsequently, VWF unravels, and recruits platelets. The presence of collagen and thrombin induces rapid platelet activation, resulting in platelet plug formation. ( **B** ) Plasmin as an alternative protease for ADAMTS-13 to cleave VWF multimers. (1) Low or reduced ADAMTS13 levels/activities result in the loss of plasma VWF processing. Under these circumstances, platelets become bound to transiently unraveled VWF, (2) leading to accumulation of VWF–platelet aggregates that occlude the microvasculature, as seen in patients presenting with TTP. (3) Endothelial cells near the occlusion sense hypoxia and present urokinase-type plasminogen activator receptors (uPAR) on their surfaces. (4) Plasminogen is activated to plasmin by uPA bound to its receptor. Plasmin then attempts to clear the obstructed vessel by cleaving VWF in the occluding thrombus.

Vascular injury induces activation of endothelial cells 29 and platelets, 30 resulting in the release of hyperreactive ultra-large (UL) VWF multimers from Weibel-Palade bodies or α-granules, respectively ( Fig. 3A ). These UL-VWF multimers are subjected to rapid proteolysis by ADAMTS13. The resulting ADAMTS13-cleaved VWF multimers are less thrombotic, but can still support normal hemostasis. 31 By inducing vasoconstriction, vascular injury also increases the hydrodynamic force, resulting in elongation of VWF multimers. 32 Unfolded VWF can bind to subendothelial collagen (via A1 and A3 domains) 26 and interact with platelets through GPIbα 33 (via VWF A1 domain 34 ) which slows down the platelets through GPIIb/IIIa (via VWF C4 domain 35 ) resulting in platelet activation, shape change, expression of activated integrins, and secretion of autocrine agents present on platelets.

### Plasmin: Candidate Protease for von Willebrand Factor

#### Rationale for Plasmin as Alternative for ADAMTS13

Deficiency in ADAMTS13 results in TTP, a disorder of thrombotic microangiopathy, characterized by an abnormal persistence of UL-VWF multimers. 36 Similar to the pathology in systemic inflammation/sepsis, thrombotic microangiopathies such as TTP are associated with acute dysfunctional endothelial cell activation, indicated by up to fourfold increased VWF antigen (VWFAg) and VWF propeptide (VWFpp). 37

Interestingly, TTP episodes occur sporadically in patients with hereditary ADAMTS13 deficiency, 38 while patients with acquired (autoantibody-mediated) ADAMTS13 deficiency can achieve clinical remission despite being severely depleted of ADAMTS13. 39 Hence, ADAMTS13 levels do not correlate with disease severity during the acute phase, suggesting the existence of regulatory mechanisms/factors other than ADAMTS13 that modulate the presentation of thrombotic microangiopathy. 39 40 However, during clinical remission, persistent ADAMTS13 deficiency is an established risk factor for clinical relapse. 41 42

Plasmin is a candidate enzyme that may serve as an alternative, or back-up, for ADAMTS13 to cleave VWF multimers. 4 The concentration of plasmin in the thrombus is above 10 nM and the environment of the thrombus protects it, at least partially, from inactivation by plasma inhibitors, most importantly α2-AP. 43 Early studies report cleavage by plasmin of the multimeric structure of VWF to lower molecular weight fragments, 44 mainly within disulfide loops, 45 resulting in loss of factor VIII 46 and VWF (ristocetin cofactor) activity. 47 An overview of reported effects of plasmin on VWF and the respective experimental concentrations used is given in Table 2 .

**Table 2:** Effects of plasmin on VWF: key findings per paper and concentrations of reagents used

Year	Investigator	Key finding(s)	Plasmin concentration used	VWF concentration used
1978	Atichartakarn et al 46	Plasmin rapidly destroys the coagulant activity of factor VIII but not ristocetin-cofactor activity.	0.6 CU/mL	0.76 mg/mL (FVIII:VWF)
1979	Henriksson and Nilsson 47	Rapid loss of VWF activity upon incubation with 1.96 CU/mL plasmin.	0.96 CU/mL; 1.92 CU/mL	2.5 U/mL (FVIII:VWF)
1979	Switzer and McKee 44	Cleavage of the multimeric structure of VWF by plasmin to lower molecular weight fragments.	0.6–0.8 CU/mL	0.6–1.2 absorbance units a
1984	Federici et al 51	Carbohydrate chains protect VWF from disaggregation secondarily to proteolytic attack by plasmin.	4 and 8 μg/mg of VWF	∼10 μg/mL b
1985	Hamilton et al 45	Plasmin degrades the large VWF multimers to smaller forms by cleaving within disulfide loops.	3.2 mg/mL (20 CU/mg)	2 mg/mL
1987	Berkowitz and Federici 53	Plasmin cleaves a 176-kD fragment from the N terminus and a 145 kDa fragment from the C terminus of the subunit. These species were demonstrated in plasmas from 4 patients with DVT treated with fibrinolytic agents, but not in type IIa VWD.	1.8 mg/mL	∼10 μg/mL b
2000	Bonnefoy and Legrand 48	VWF is rapidly released from native subendothelium when incubated with plasmin on a confluent endothelial cell monolayer. The released VWF is more resistant to proteolysis than constitutively secreted VWF.	0.2 CU/mL (200 nM)	6 μg/mL
2010	Tanka-Salamon et al 49	At its physiological concentration VWF is able to protect fibrinogen from degradation by plasmin.	12.5 nM	10 μg/mL
2012	Wohner et al 50	Plasmin at concentrations of in vivo relevance resulted in extensive degradation of VWF within several minutes.	50 nM	10 μg/mL
2014	Tersteeg et al 4	Efficiency of VWF cleavage by plasmin is a function of its conformation: plasmin has limited affinity for binding to, and cleavage of, globular VWF, but shear- or denaturant-mediated unfolding strongly enhances this process.	Not specified c	Not determined d
2017	Brophy et al 3	Globular VWF is resistant to plasmin cleavage under static conditions, but is readily cleaved by plasmin under shear Plasmin cleaves the K1491-R1492 peptide bond within the VWF A1–A2 linker region. VWF susceptibility to plasmin proteolysis is modulated by local N-linked glycan expression within A1A2A3, and can be specifically inhibited by heparin binding to the A1 domain.	12.8 nM	10 μg/mL

Abbreviations: CU, caseinolytic units; VWF, von Willebrand factor.

a VWF concentration was estimated by the absorbance at 280 nm, corrected for light scattering, and expressed as absorbance units.

b Estimated plasma concentration of VWF.

c 216 μg/mL plasminogen + 10 ng/mL uPA or 10 U/mL streptokinase.

d VWF from cultured human umbilical vein endothelial cells (HUVECs) stimulated to release VWF by addition of phorbol 12-myristate 13-acetate (PMA).

#### Mechanisms of VWF Proteolysis by Plasmin

Plasmin, locally produced during fibrinolysis, can release VWF within minutes from native subendothelium. Thereby, proteolytic fragments are generated with potential activity in terms of platelet aggregation. 48 VWF is a relatively poor plasmin substrate compared with fibrinogen. In fact, VWF protects fibrinogen (but not fibrin) against degradation by plasmin, thereby preserving procoagulant activity in plasma and its adhesive role in platelet thrombi. 49 This might indicate that the presence of VWF in platelet-rich thrombi may contribute to their resistance to thrombolytic therapy. 49

Plasmin has limited affinity for binding to and cleavage of globular VWF, but shear- or denaturant- (e.g., ristocetin) induced unfolding and consequent exposure of the VWF A1 domain strongly enhance this process. 3 4 50 Of note, plasmin-binding sites and plasmin proteolysis sites within VWF are distinct. Under shear stress, conformational changes in A1A2 facilitate plasmin binding to lysine-rich regions 1405 ^KKKK^ 1408 in the VWF A1 domain, which subsequently enables specific plasmin cleavage at the K1491-R1492 peptide bond within the VWF A1-A2 linker region. 3 4

VWF glycans play an important role in modulating VWF cleavage by plasmin. 3 51 First, desialylation of VWF enhances proteolysis by plasmin while it inhibits ADAMTS13 proteolysis. 3 52 53 Second, susceptibility of VWF to plasmin proteolysis at K1491-R1492 is modulated by local N-linked glycan expression within A1A2A3. More precisely, N-linked glycans expressed at N1515 and N1574 within the A2 domain protect VWF against proteolysis by both plasmin and ADAMTS13. 3 54 55 Finally, ABO(H) blood group determinants modulate VWF susceptibility to proteolysis by ADAMTS13 (O ≥ B > A ≥ AB) but do not influence plasmin-mediated cleavage. 3 56

#### Disaggregation of VWF–platelet Complexes in the Presence of Plasmin

In the complete absence of ADAMTS13, as in TTP, platelets as well as UL-VWF are present in the circulation ( Fig. 3B ). However, obstructive microthrombi do not form spontaneously until a triggering event, for instance an infection, occurs. At that moment, vascular endothelial cells are the first to react on differences in oxygenation and respond to hypoxia through expression of extracellular uPA receptor. 57 Subsequently, uPA-dependent plasmin formation on endothelial cells results in degradation of platelet–VWF complexes. 4 Furthermore, exogenously added thrombolytic agents degrade platelet–VWF complexes with equal efficacy and can be securely controlled by the administration of lysine analogues. 4 This may have important clinical implications, as thrombolytic agents may thus have therapeutic value in the treatment of TTP. 4 In fact, Tersteeg et al found that a single dose (10 U/mL, corresponding to ∼20% of the loading dose given for pulmonary embolism) of the plasminogen (human, injected at 20 mg/kg) activator streptokinase was sufficient to attenuate symptoms of TTP and correct the thrombocytopenia in an ADAMTS13-deficient mouse model. Importantly, no evidence of bleeding or a perturbed secondary hemostasis was observed after this treatment. 4 Hence, further studies on the safety and efficacy of therapeutic application of thrombolytic agents for TTP are warranted.

## Plasmin and Platelets

A comprehensive overview of platelet adhesion, activation, and aggregation was reviewed by Broos et al. 58 Key findings on the effects of plasmin on platelets reported in literature and the experimental concentrations of plasmin used are summarized in Table 3 .

**Table 3:** Effects of plasmin on platelets: key findings per paper and concentrations of reagents used

Year	Investigator	Key finding(s)	Plasmin concentration used
1973	Niewiarowski et al 67	Plasmin-induced platelet aggregation is reversible and is accompanied by significant granule release.	2.2 CU/mg
1985	Adelman et al 91	Treatment of washed platelets with plasmin resulted in progressive loss of GPlb accompanied by loss of the agglutination response when combined with ristocetin in the presence of VWF.	1 CU/mL
1985	Adelman et al 92	Plasmin reduced ristocetin-mediated agglutination of washed platelets in the presence of VWF following a 60-min incubation. Plasmin treatment of washed platelets released a glycocalicin-related antigen into the surrounding medium, corresponding to loss of VWF-dependent, ristocetin-induced agglutination.	0.05–1.0 CU/mL
1985	Schafer and Adelman 79	Plasmin concentrations that did not affect platelet shape change, release, or aggregation (< 1.0 CU/mL) caused a dose- and time-dependent inhibition of platelet aggregation in response to thrombin, ionophore A23187, and collagen. Complete loss of aggregation occurred at 0.1–0.5 CU/mL of plasmin.	<1.0 CU/mL 0.1–0.5 CU/mL
1986	Schafer et al 70	In washed human platelets, plasmin at concentrations of ≥ 1.0 CU/mL induces aggregation.	≥ 1.0 CU/mL
1991	Cramer et al 95	Plasmin treatment of platelets at 37°C resulted in the disappearance of GPlb from the cell surface and its subsequent redistribution into the channels and vesicles of the surface-connected canalicular system.	0.2 CU/mL
1991	Lu et al 78	Lowering the temperature from 37 to 22°C, plasmin at low concentrations (0.1–0.5 CU/mL) fully activated platelets. When platelets were treated with 0.2 CU/mL of plasmin, lowering the temperature resulted in increased expression of fibrinogen receptors, in platelet release and aggregation.	0.1–0.5 CU/mL
1992	Gouin et al 84	Incubation of human platelet-rich plasma with streptokinase does not produce any detectable platelet activation but leads to a time-dependent inhibition of ADP-induced aggregation accompanied by substantial fibrinogenolysis.	Not determined a
1994	Pasche et al 83	Plasmin treatment reduced maximal reversible fibrinogen binding in a dose-dependent fashion, and this reduction in binding was accompanied by a correlative reduction in the maximal rate of aggregation.	0.4–4 CU/mL
1995	Loscalzo et al 74	Plasmin at higher concentrations (∼1 CU/mL), plasmin activates the platelet directly.	1.0 CU/mL
1995	Nakamura et al 71	Plasmin produces a small rise in platelet cytosolic Ca ^2+^ and a tyrosine kinase-dependent enhancement of Ca ^2+^ turnover.	≥ 1.0 CU/mL
1997	Kinlough-Rathbone et al 80	Incubation with plasmin almost completely inhibited thrombin-induced aggregation, release of serotonin, and increase in cytosolic Ca ^2+^ .	0.2 CU/mL
2000	Ishii-Watabe et al 72	Plasmin causes the degranulation of platelets; subsequently, ADP released from granules plays a crucial role in the induction of platelet aggregation.	≥ 1.0 CU/mL
2001	Ervin and Peerschke 77	Sustained exposure (60 min) of platelets to very low plasmin doses leads to platelet activation, both at 22 and 37°C. The resulting platelet aggregation was not accompanied by dense or α-granule secretion.	0.05 CU/mL
2004	Quinton et al 88	Desensitization of PAR1 has no effect on plasmin-induced platelet aggregation. PAR4 is cleaved by plasmin at the thrombin-cleavage site R47. Desensitization of PAR4 completely eliminates plasmin-induced aggregation. Platelets treated with a PAR4 antagonist do not aggregate in response to plasmin.	1 CU/mL

Abbreviations: CU, caseinolytic units; VWF, von Willebrand factor.

a 300 IU/mL streptokinase was added to platelet-rich plasma.

### (In)activation of Platelets by Plasmin

Under physiological conditions, plasmin activity is restricted to the proximity of the thrombus by plasma proteinase inhibitors. In stroke or deep vein thrombus formation, treatment with thrombolytic drugs can lead to free active plasmin in the circulation. 59 60 Sometimes, after a successful reperfusion, reocclusion of the damaged vessel occurs and marked platelet activation can be detected. 20 61 62 63 64 65 66

In the early 1970s, Niewiarowski et al were the first to report reversible plasmin-induced platelet aggregation, accompanied by a significant granule release. 67 68 In contrast, plasmin treatment could also reduce platelet aggregation after stimulation with thrombin, collagen, or adenosine diphosphate (ADP). 67 69 These results already suggested a dual effect of plasmin depending on plasmin concentration, temperature, exposure time, and platelet environment.

Plasmin, at concentrations ≥1 caseinolytic unit [CU]/mL causes platelet degranulation and aggregation in in vitro studies using isolated, plasma-free platelets. 61 70 71 72 73 74 To compare, the amount of plasminogen in 1 mL of normal plasma corresponds to 3 to 4 CU, 75 which amounts to a concentration of 20.3 ± 2.6 mg/100 mL or 80 µM of plasmin. The clinical dose of r-tPA used for treatment of ischemic stroke (0.9 mg/kg for 1 hour) resulted in the generation of 1.6 CU/mL plasmin in a canine animal model. 76 More in detail, plasmin activates protein kinase C (PKC) and stimulates dose-dependent diacylglycerol (and phosphatic acid) production by phospholipase C activation. 70 Subsequently, plasmin produces a small rise in platelet cytosolic Ca ^2+^ and a tyrosine kinase-dependent enhancement of Ca ^2+^ turnover. 70 71 Upon plasmin-induced platelet degranulation, ADP most likely plays a crucial role in the induction of platelet aggregation via the P2Tac receptor. 72 Interestingly, prolonged (>60 minutes) exposure of platelets to very low doses (0.05 CU/mL) of plasmin also leads to platelet aggregation and enhanced expression of procoagulant activity. 77 Likewise, upon lowering the temperature to 22°C, incubation with low concentrations of plasmin (<0.1 CU/mL) induces strong platelet aggregation. 78 These results may be of clinical relevance, because the fibrinolytic system was found to be activated during cardiopulmonary bypass, in which the temperature of the patient's blood is reduced. 78

Plasmin at concentrations less than 1.0 CU/mL can cause a dose- and time-dependent inhibition of platelet aggregation and thromboxane B2 (TXB _2_ , inactive metabolite of TXA _2_ ) production in response to thrombin, ionophore A23187, and collagen. 61 74 79 80 This plasmin-induced inhibitory effect may result from a proteolysis-mediated increase in cyclic AMP, 81 blockage of the mobilization of arachidonic acid from membrane phospholipid pools, 79 and/or modification of the expression of glycoprotein receptors (GPIb and GPIIb/IIIa, see below for more details). Interestingly, plasmin synergizes with prostaglandin I2 in inhibiting thrombin- and ADP-induced platelet activity, a finding of potential importance for the control of platelet accrual at sites of vascular injury, where the release of tPA and prostaglandin I2 by adjacent endothelial cells is generally increased. 82

Of note, in plasma, the main effect of plasmin is impairment of aggregation. 83 84 However, this is not related to a direct effect of plasmin on platelets. Plasmin degrades fibrinogen and fibrin/fibrinogen degradation products bind to fibrinogen and/or GPIIb/IIIIa receptors, both resulting in a reduction of the formation of fibrinogen bridges between platelets. 85 Additionally, in plasma, plasmin is inhibited by α2-antiplasmin. 86

#### Plasmin Activates Platelets via PAR4

Plasmin cleaves both thrombin receptors PAR1 and PAR4 on platelets. 87 88 Cleavage by plasmin was reported to be 1,700-fold lower relative to thrombin cleavage. 89 90 Four cleavage sites have been identified on the PAR1 exodomain (R41, R70, K76, and K82). R41 is the thrombin-cleavage site and generates a transiently activated exodomain. The R70, K76, and K82 sites are located on a linker region that tethers the ligand to the body of the receptor. However, the high affinity of plasmin for R70, K76, and K82 and the low affinity for R41 (thrombin-cleavage site) result predominantly in inactivation of PAR1 by truncating the tethered ligand at the distal sites. 87 Additionally, desensitization of PAR1 had no effect on plasmin-induced platelet aggregation. 88 PAR4, on the other hand, is cleaved by plasmin at the thrombin-cleavage site R47. 88 As a result, desensitization of PAR4 completely eliminated aggregation in response to plasmin, and platelets treated with a PAR4 antagonist, as well as platelets isolated from PAR4 homozygous null mice, failed to aggregate in response to plasmin. Altogether, Quinton et al concluded that plasmin induces platelet aggregation primarily through slow cleavage of PAR4. 88

#### Plasmin Induces Reversible Translocation of Platelet GPIb

The addition of plasmin to washed platelets results in progressive loss of GPIb as measured by fluorescence flow cytometry and by the loss of ristocetin-induced VWF-dependent platelet agglutination. 91 92 The loss of platelet surface GPIb, initially assigned to plasmin-mediated cleavage of GPIb, 92 93 94 is now known to be related to a redistribution of GPIb, which moves from the external membrane into the surface-connected canalicular system. 95 This translocation of GPIb is related to actin polymerization, as cytochalasin D (a specific inhibitor of actin polymerization) inhibited the migration of GPIb after plasmin stimulation. 95 Subsequently, neutralization of plasmin by the inhibitors aprotinin and tripeptide Val-Phe-Lys-CH _2_ Cl permitted a time-dependent recovery of platelet agglutination. 96 The functional recovery was accompanied by a restoration of a normal amount of GPIb on the platelet surface. This suggests a reverse translocation of the GPIb molecules initially present at the cell surface. 96

#### Effect of Plasmin on GPIIb/IIIa

Plasmin exerts opposing effects on GPIIb/IIIa, resulting in enhanced or impaired fibrinogen binding and platelet aggregation. On the one hand, plasmin-induced platelet activation and granule release result in the expression of active GPIIb/IIIa receptors on the cell surface. 97 On the other hand, in plasma, plasmin degrades GPIIIa by cleaving a proteolytically susceptible loop domain, but only after degradation of the solubilized GPIIb/IIIa by *Staphylococcus aureus* V8 (Glu-C) endoprotease. 83 In contrast, treatment of isolated platelets with plasmin has no effect on GPIIb/IIIa expression. 95 These findings suggest that, in plasma, plasmin modifies GPIIIa by a unique proteolytic event that is dependent on fibrinogen binding and, consequently, is accompanied by significant reduction in fibrinogen binding and aggregation. 83

## Conclusion

Although the mechanism of plasmin generation and its function in fibrinolysis are rather well-defined, the precise (patho)physiological effects of plasmin on other components of the coagulation cascade are hitherto not completely understood. In this review, we provide an overview of the available evidence on the effects of plasmin on VWF and platelets. Summarizing, it can be stated that, in addition to ADAMTS13, plasmin can regulate the thrombogenicity of VWF through its protease activity. This role of plasmin appears particularly important in pathologies in which high-molecular-weight VWF multimers accumulate, as exemplified by TTP. Moreover, although plasmin-mediated platelet activation is unlikely to play a significant role in normal hemostasis, during pharmacological thrombolysis where the plasmin activity could reach as high as 1 CU/mL, it may have implications in terms of vessel patency and effectiveness of treatment. Hence, future research should focus on obtaining a better understanding of the molecular mechanisms of plasmin binding to and cleavage of VWF as well as of plasmin's effects on platelet activation and aggregation. For experiments with platelets, particular attention should be given to simulating the in vivo environment as much as possible in the experimental conditions to obtain reliable results. Together, this knowledge on the interaction of plasmin, VWF, and platelets will support the ongoing development of novel therapies 98 99 as well as the use of existing thrombolytic therapy to potentially prevent the occurrence of, or degrade, existing VWF–platelet aggregates in conditions such as TTP.
